# Convergence of MCR-8.2 and Chromosome-Mediated Resistance to Colistin and Tigecycline in an NDM-5-Producing ST656 *Klebsiella pneumoniae* Isolate From a Lung Transplant Patient in China

**DOI:** 10.3389/fcimb.2022.922031

**Published:** 2022-07-11

**Authors:** Jiankang Zhao, Ziyao Li, Yulin Zhang, Xinmeng Liu, Binghuai Lu, Bin Cao

**Affiliations:** ^1^ Laboratory of Clinical Microbiology and Infectious Diseases, Department of Pulmonary and Critical Care Medicine, National Center for Respiratory Medicine, Center of Respiratory Medicine, National Clinical Research Center for Respiratory Diseases, China-Japan Friendship Hospital, Beijing, China; ^2^ Institute of Respiratory Medicine, Chinese Academy of Medical Sciences, Peking Union Medical College, Beijing, China; ^3^ Tsinghua University-Peking University Joint Center for Life Sciences, Beijing, China; ^4^ Department of Respiratory Medicine, Capital Medical University, Beijing, China

**Keywords:** *Klebsiella pneumoniae*, *bla*_NDM-5_, *mcr-8.2*, colistin, tigecycline

## Abstract

We characterized the first NDM-5 and MCR-8.2 co-harboring ST656 *Klebsiella pneumoniae* clinical isolate, combining with chromosomal gene-mediated resistance to colistin and tigecycline. The *K. pneumoniae* KP32558 was isolated from the bronchoalveolar lavage fluid from a lung transplant patient. Complete genome sequences were obtained through Illumina HiSeq sequencing and nanopore sequencing. The acquired resistance genes and mutations in chromosome-encoded genes associated with colistin and tigecycline resistance were analyzed. Comparative genomic analysis was conducted between *mcr-8.2*-carrying plasmids. The *K. pneumoniae* KP32558 was identified as a pan-drug resistant bacteria, belonging to ST656, and harbored plasmid-encoded *bla*_NDM-5_ and *mcr-8.2* genes. The *bla*_NDM-5_ gene was located on an IncX3 type plasmid. The *mcr-8.2* gene was located on a conjugative plasmid pKP32558-2-mcr8, which had a common ancestor with another two *mcr-8.2*-carrying plasmids pMCR8_020135 and pMCR8_095845. The MIC of KP32558 for colistin was 256 mg/L. The *mcr-8.2* gene and mutations in the two-component system, *pmrA* and *crrB*, and the regulator *mgrB*, had a synergistic effect on the high-level colistin resistance. The truncation in the *acrR* gene, related to tigecycline resistance, was also identified. *K. pneumoniae* has evolved a variety of complex resistance mechanisms to the last-resort antimicrobials, close surveillance is urgently needed to monitor the prevalence of this clone.

## Introduction

The emergence and worldwide dissemination of carbapenem-resistant *Klebsiella pneumoniae* (CRKP) have posed a great threat to public health (Nordmann et al., 2009). The main resistance mechanism for CRKP is the production of carbapenemases, of which New Delhi metallo-β-lactamase (NDM) is capable of hydrolyzing all commonly-used β-lactams except monobactams. The *bla*_NDM-5_ gene was first identified from an *Escherichia coli* isolate in 2011 from a patient with a hospitalization history in India (Li et al., 2018). Afterward, NDM-5-producing *K. pneumoniae* isolates have been documented worldwide (Rojas et al., 2017; Li et al., 2018; Zhao et al., 2021). Compared to NDM-1, NDM-5 has two amino acid substitutions at Val88Leu and Met154Leu, showing increased resistance to carbapenems and broad-spectrum cephalosporins when expressed under its native promoter (Hornsey et al., 2011).

For infections caused by NDM-5-producing CRKP, tigecycline and colistin are the last treatment options (Li et al., 2021; Xu et al., 2021b). In recent years, colistin resistance in *Enterobacterales* isolates has been found more frequently. Several molecular mechanisms have been associated with colistin resistance, such as chromosomal mutations in the genes that encode PmrA/PmrB, PhoP/PhoQ and CrrA/CrrB two-component systems (TCS), and inactivation of the regulator MgrB (Cannatelli et al., 2013; Mcconville et al., 2020). Since the first plasmid-encoded colistin resistance gene, *mcr-1*, was identified in *E. coli* in 2015 (Liu et al., 2016), nine variants of *mcr-1* (*mcr-2* up to *mcr-10*) have also been identified in *Enterobacterales* (Elbediwi et al., 2021). The horizontal transferability of plasmids facilitates the rapid dissemination of colistin resistance. Furthermore, increasing consumption of tigecycline has been concurrent with increasing reports of tigecycline resistance (Osei Sekyere et al., 2016). The main mechanism is the overexpression of resistance-nodulation-cell division (RND)-type efflux pumps (*acrAB* and *oqxAB*) and mutations in efflux pump regulatory genes (*ramA* and *acrR*) and regulatory genes of the SoxS and MarAB (*soxR* and *marR*) (He et al., 2015; Osei Sekyere et al., 2016; Xu et al., 2021a).

*K. pneumoniae* strains, presenting with extensive resistance to carbapenems, tigecycline and colistin simultaneously, would pose a great threat to public health. In this study, we isolated a pan-drug resistant *K. pneumoniae* strain from the bronchoalveolar lavage fluid (BALF) specimen of a lung transplant patient. Revealing its mechanism of drug resistance will play a positive role in preventing the spread of this strain.

## Materials and Methods

### *K. pneumoniae* Isolates and the Reference Plasmids

Two *K. pneumoniae* isolates (KP31166 and KP32558) were recovered from the BALF specimens of a lung transplant patient 1 and 4 months after surgery, respectively. For comparative genomic analysis, ten *mcr-8.2-*harboring plasmids were collected from the NCBI genome database, including plasmids pMCR8_020135 (CP037964), pMCR8_095845 (CP031883), pVNCKp115 (LC549807), pZZW20-88K (CP058962), p2019036D-mcr8-345kb (CP047337), pD120-1_83kb (CP034679), pVNCKp83 (LC549808), pSCKLB555-4 (CP043936), p2018C01-046-1_MCR8 (CP044369) and p18-29mcr-8.2 (MK262711).

### Antimicrobial Susceptibility Testing

*In vitro* susceptibility tests of amikacin, tobramycin, minocycline, doxycycline, ceftazidime, cefepime, piperacillin/tazobactam, cefoperazone/sulbactam, aztreonam, imipenem, meropenem, levofloxacin, ciprofloxacin, and sulfamethoxazole/trimethoprim were performed using the Vitek-2 system in N335 susceptibility cards (bioMérieux, France). The minimum inhibitory concentrations (MICs) of tigecycline and colistin were determined using the microdilution broth method (bio-KONT, Ltd. China). The production of carbapenemases was determined using the modified carbapenem inactivation method (mCIM) and EDTA-modified carbapenem inactivation method (eCIM) as recommended by the Clinical Laboratory Standards Institute (CLSI) (Clinical and Laboratory Standards Institute, 2019). The breakpoints of tigecycline and colistin were defined by the European Committee on Antimicrobial Susceptibility Testing (EUCAST, version 11.0, http://www.eucast.org/). In particular, MICs > 0.5 mg/L and > 2 mg/L will be defined as resistant to tigecycline and colistin, respectively.

### Hypermucoviscous Phenotype Determination

The string test was used to identify the hypermucoviscous phenotype of *K. pneumoniae* strains. Strains exhibiting a viscous string >5 mm were considered hypermucoviscous (Shon et al., 2013).

### Whole-Genome Sequencing and Annotation

Complete genome sequencing was carried out using Illumina HiSeq 2500 platform (for both KP32558 and KP31166) as well as nanopore sequencing method on MinION flow cells (for KP32558 only). Raw reads were filtered to remove low-quality sequences and adaptors using skewer (Jiang et al., 2014) and PoreChop (https://github.com/rrwick/Porechop), respectively. *De novo* assembly was conducted using SPAdes Genome Assembler v3.13.1 (Prjibelski et al., 2020) and Unicycler (Wick et al., 2017). Gene prediction for 11 genomes, including one from this study and 10 from the NCBI genome database, was performed using Prokka 1.12 (Seemann, 2014). Genomic islands were predicted using IslandViewer 4 (Bertelli et al., 2017). Insertion sequences were identified using the ISfinder database (Siguier et al., 2006). The antimicrobial resistance genes, multilocus sequence types (MLST) and plasmid replicon were analyzed *via* the CGE server (https://cge.cbs.dtu.dk/services/). Virulence genes were identified using the BIGSdb *Klebsiella* genome database (http://bigsdb.Pasteur.fr/klebsiella/klebsiella.html). The single nucleotide polymorphism (SNP) was called using Snippy (https://github.com/tseemann/snippy). The heatmap was generated using an in-house R script.

### Phylogenetic Analysis

A total of 11 *mcr-8.2*-carrying plasmids were included and aligned to reference plasmid pKP32558-2-mcr8 (GenBank accession no. CP076032, this study) using Snippy. A SNP maximum likelihood (ML) tree was constructed by using FastTree 2 (Stamatakis, 2014) with a general time reversible (GTR) model of nucleotide substitution and a gamma distribution of rate heterogeneity. Phylogenetic clusters were identified using hierarchical Bayesian analysis of population structure, in which the first level of clustering was used to define clades (Cheng et al., 2013). The phylogenetic tree was annotated by using FigTree v1.4.3 (http://tree.bio.ed.ac.uk/software/figtree/).

### Plasmid Conjugation Assays

The plasmid conjugation experiment was performed for *K. pneumoniae* strain KP32558, and azide-resistant *E. coli* J53 was used as the recipient strain. Transconjugants were selected on LB plates containing azide (100 mg/L) and meropenem (2 mg/L) or colistin (2 mg/L). Antimicrobial susceptibility testing, PCR amplification and S1-PFGE were performed to confirm the successful transfer. The transfer frequency was calculated as the number of transconjugants per recipient.

### Assessment of Gene Expression by Quantitative Real-Time PCR

Total RNA was isolated from the *K. pneumoniae* strains using the RNeasy minikit (Qiagen), according to the manufacturer’s instructions. The expression of the *acrB*, *acrR*, *crrA*, *crrB*, *crrC*, *phoP*, *phoQ*, *pmrA, pmrB, pmrC*, *pmrD*, *pmrK*, *mgrB*, and *rpoB* genes was measured using qRT-PCR on an Applied Biosystems QuantStudio 5 real-time PCR system (ThermoFisher Scientific). The expression levels of the target genes were normalized to the *rpoB* gene using ΔΔ*C_T_* method, and the strain *K. pneumoniae* ATCC 13883 was used as reference. The primers used for qRT-PCR are listed in **Table S1**. All tests were performed three times.

### Growth Kinetics

Growth curves for the recipients *E. coli* J53 and transconjugants were measured as follows: LB broths containing 1 × 10^6^ CFU/mL bacteria were incubated overnight at 37°C with consecutive shaking. Meanwhile, the same broth without bacteria was set as the growth control. OD600 measurement was taken at 0, 2, 4, 6, 8, 10, and 12 h to construct a growth curve. All experiments were performed in duplicate. Statistically significant differences in growth kinetics including slope and time analysis were tested by using the R package “statmod” (Giner and Smyth, 2016). A *P* value of 0.05 or less was defined as significant.

## Results

### Clinical Data of the Patient

A lung transplant patient was given colistin for two days on the 3^rd^ day after the surgery for the prevention of infections. The *K. pneumoniae* isolates KP31166 and KP32558 were detected in his BALF specimens approximately one and four months later, respectively. In addition, the patient had normal blood routine and C-reactive protein test results and had no fever when *K. pneumoniae* was detected. Considering the condition of the patient and antimicrobial susceptibility results of the *K. pneumoniae*, no specific anti-infective treatment was given. However, *K. pneumoniae* was not isolated from BALF specimens since then, and the patient recovered well.

### General Phenotypic and Genotypic Characteristics

According to the antimicrobial susceptibility testing, strains KP32558 and KP31166 were all CRKP and were resistant to all tested antibiotics (**Table 1**). Both strains exhibited the same MICs for tigecycline (4 mg/L) and colistin (256 mg/L). ECIM and mCIM tests indicated that the two strains produced metallo-β-lactamase. Strains KP32558 and KP31166 carried the same resistance genes, and only two SNPs were found between them based on whole-genome SNP analysis (data not shown). According to a previous criterion (Schurch et al., 2018), the two strains were considered homologous and derived from the same ancestor.

**Table 1:** Antibiotic resistance characteristics (MICs, mg/L) of 4 clinical *K. pneumoniae* strains and the transconjugant of KP32558.

Antibiotics	KP31166	KP32558	J53	Transconjugant 1 (MCR-8.2)	Transconjugant 2 (NDM-5)
Amikacin	>=64	>=64	<=2	<=2	4
Tobramycin	>=16	>=16	<=1	4	<=1
Doxycycline	>=16	>=16	2	>=16	2
Minocycline	>=16	>=16	2	8	2
Tigecycline^a^	4	4	0.25	0.25	0.25
Cefepime	>=32	>=32	<=0.12	<=0.12	16
Ceftazidime	>=64	>=64	<=0.12	<=0.12	>=64
TZP	>=128	>=128	<=4	<=4	>=128
SCF	>=64	>=64	<=8	<=8	>=64
Aztreonam	>=64	>=64	<=1	<=1	<=1
Imipenem	>=16	>=16	<=0.25	<=0.25	>=16
Meropenem	>=16	>=16	<=0.25	<=0.25	>=16
Levofloxacin	>=8	>=8	<=0.12	1	<=0.12
Ciprofloxacin	>=4	>=4	<=0.25	0.5	<=0.25
Colistin^a^	256	256	1	4	1
SXT	>=320	>=320	<=20	>=320	<=20

a MIC of tigecycline and colistin was detected using microdilution broth method. SAM, Ampicillin/sulbactam; TZP, Piperacillin/tazobactam; SCF, Cefoperazone/sulbactam; SXT, Sulfamethoxazole/trimethoprim.

Strain KP32558 was randomly selected for further analysis, which was not hypermucoviscous and assigned to ST656. Neither *rmpA*/*rmpA2* genes nor *iucABCDiutA* and *iroBCDN* virulence gene clusters were found in this strain. Resistance gene annotation showed that about 40 resistance genes were located on its plasmids (**Table S2**).

### Plasmid Containing *bla*_NDM-5_ Gene

The *bla*_NDM-5_ gene located in the 5^th^ plasmid pKP32558-5-ndm5 (46161 bp in size, GenBank accession number CP076035), which belonged to the IncX3 group and did not harbor other resistance genes. Microbial nucleotide search for GenBank showed that pKP32558-5-ndm5 was nearly identical to a series of *bla*_NDM-5_ -carrying plasmids with the same size in length. Specifically, pKP32558-5-ndm5 showed 100% identity and coverage with plasmid pCREC-591_4 (CP024825, 46161 bp) from *E. coli* strain CREC-591, and 100% identity and 99.8% coverage with the plasmid pNDM_MGR194 (KF220657, 46253 bp), which was a typical *bla*_NDM-5_-carrying plasmid recovered from a *K. pneumoniae* isolate in India (Krishnaraju et al., 2015). Moreover, we found a genomic island region in pKP32558-5-ndm5, the *bla*_NDM-5_ gene and its flanking contents were also located in this region.

Similar to other *bla*_NDM-5_-carrying plasmids, several conjugal transfer genes (such as *virD4*, *virB4*, *virB8* and *virB9*) were identified in the plasmid pKP32558-5-ndm5. Conjugation experiment showed that this plasmid could be successfully transferred to the recipient *E. coli* J53 as reported previously (Zhao et al., 2021) at a frequency of 10^-4^ (transconjugant/recipient), and the transconjugant displayed resistance to imipenem and meropenem (**Table 1**).

### Presence of *mcr-8* Gene in a Two-Replicons Plasmid

**Figure 1:**
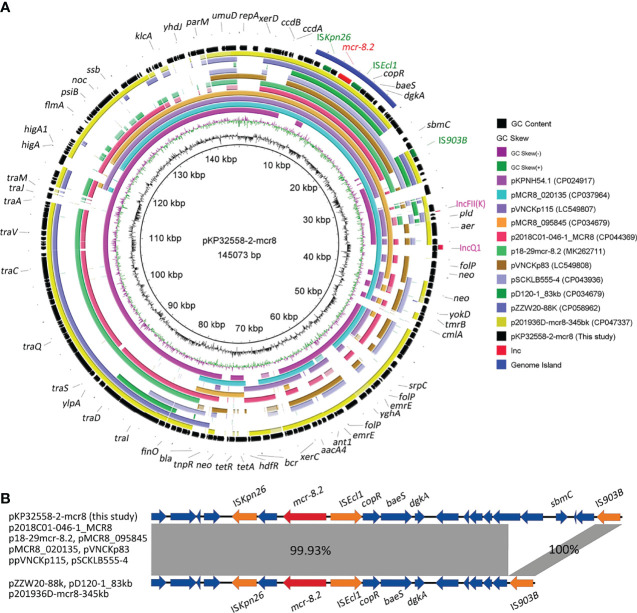
Genetic features of the *mcr-8.2*-carrying plasmid in *K. pneumoniae* strain KP32558. **(A)** Circular map of plasmid pKP32558-2-mcr8 and its reference plasmids. The outer blue circle is the genomic island region of plasmid pKP32558-2-mcr8. Red text on the plasmid map indicates *mcr-8.2* gene, the green text indicates insertion sequences around *mcr-8.2*, and pink text represents the replicons. **(B)** Genetic contents of *mcr-8.2* gene. Coding sequences are indicated by arrows. Sequences of shared homology between two plasmids are marked by gray shading.

An *mcr-8.2* gene (1698 bp) was found in the second plasmid pKP32558-2-mcr8 (CP076032) by blasting against the resfinder database. Microbial nucleotide search for GenBank suggested that pKP32558-2-mcr8 had the highest alignment score with the plasmid pKPNH54.1, which was derived from *K. pneumoniae* strain NH54, and did not contain the *mcr* gene. Comparative genomic analysis was conducted among pKP32558-2-mcr8, pKPNH54 and other 10 *mcr-8.2*-containing plasmids obtained from the NCBI database. The result showed that these plasmids were not identical to each other (**Figure 1A**), and the plasmids pKPNH54.1 (CP024917), pMCR8_020135 (CP037964) and pVNCKp115 (LC549807) could be aligned to different parts of pKP32558-2-mcr8. Despite the differences between these *mcr-8.2*-carrying plasmids, the commonality between them was that they had identical genetic environments around the *mcr-8.2* gene, IS*Kpn26*-orf-*mcr-8.2*-IS*Ecl1*-*copR-baeS-dgkA* (**Figure 1B**). Besides, plasmid pZZW20-88k, pD120-1_83kb and p201936D-mcr8-345kb had a short fragment deletion in front of IS*903B* compared to other plasmids. Of note, all these genes were located on a genomic island region.

**Figure 2:**
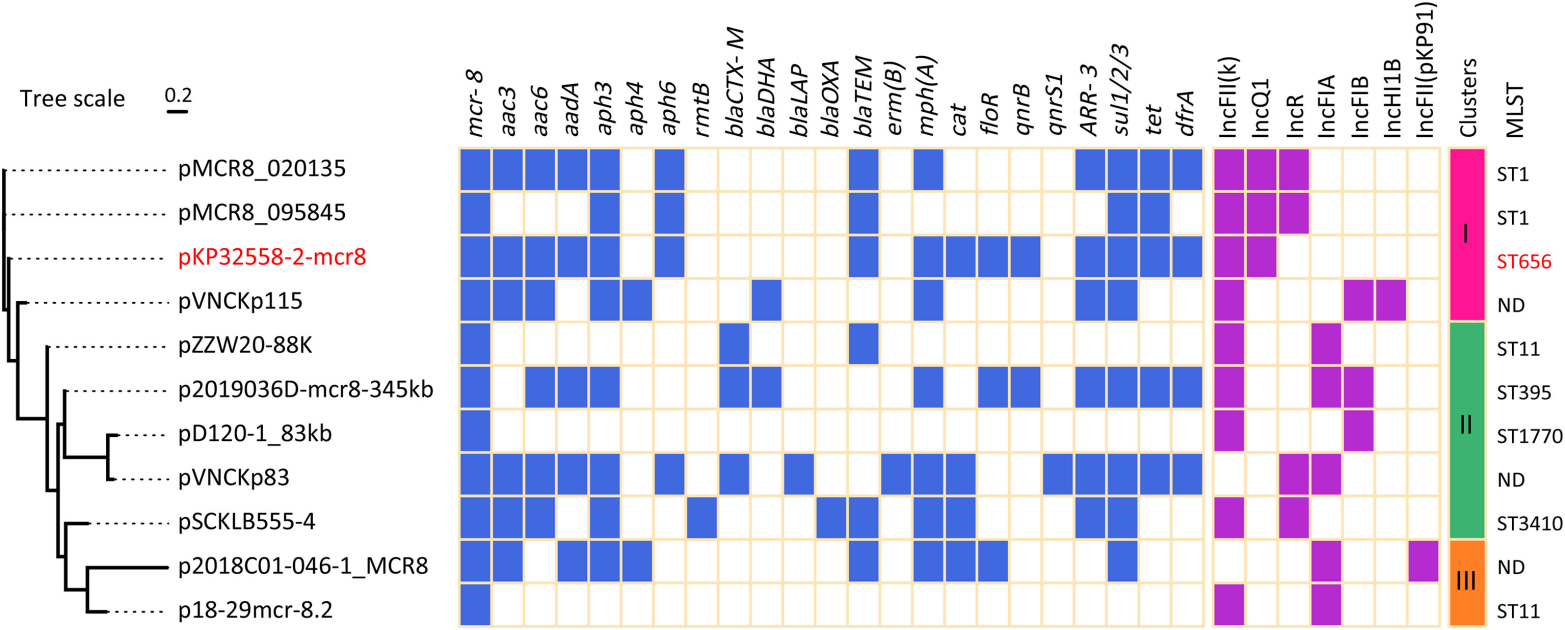
Resistance genes and replicons of *mcr-8.2*-carrying plasmids. The pKP32558-2-mcr8 (CP CP076032) was derived from *K. pneumoniae* KP32558 of this study. The genomic sequences of plasmids pMCR8_020135 (CP037964), pMCR8_095845 (CP031883), pVNCKp115 (LC549807), pZZW20-88K (CP058962), p2019036D-mcr8-345kb (CP047337), pD120-1_83kb (CP034679), pVNCKp83 (LC549808), pSCKLB555-4 (CP043936), p2018C01-046-1_MCR8 (CP044369) and p18-29mcr-8.2 (MK262711) were obtained from NCBI database.

Plasmid typing suggested that these *mcr-8.2*-carrying plasmids contained 7 different replicons, and IncFII(K) replicon was found in most plasmids (9/11) (**Figure 2**). In addition to IncFII(K) replicon, the plasmid pKP32558-2-mcr8 also had a truncated IncQ1 type replicon, which was also present in pMCR8_020135 and pMCR8_095845. The two plasmids were derived from different hospitals in China. The phylogenetic tree showed that these plasmids were clustered into 3 major clades. Plasmid pKP32558-2-mcr8, pMCR8_020135, pMCR8_095845 and pVNCKp115 belonged to the same clade.

### Resistance Gene and Conjugation Experiment of Plasmid pKP32558-2-mcr8

Resistance genes were identified using the resfinder database. In addition to *mcr-8.2*, several other resistance genes were found in plasmid pKP32558-2-mcr8, including *aac*, *aad* and *aph* for aminoglycoside, *bla*_TEM_ for β-lactam, *mph(A)* for macrolide, *arr-3* for rifampicin, *sul1/2/3/*for sulphonamide, *tet* for tetracycline and *dfrA* for trimethoprim antibiotics (**Figure 2**). Conjugation experiments showed that the plasmid pKP32558-2-mcr8 could be transferred into *E. coli* J53 at a frequency of 10^-7^.

### MLST of *mcr*-8.2-Carrying Strains

MLST analysis suggested that both KP31166 and KP32558 in this study belonged to ST656, and the other 10 *mcr*-8.2-carrying strains belonged to different ST types, including ST1, ST11, ST395, ST1770 and ST3410. Among them, ST1, ST656 and ST3410 belonged to the same clonal complex, and there was only one allele difference between them. For three isolates, their chromosomal sequences were not deposited in GenBank, therefore the MLST results could not be obtained (**Figure 2**).

### Chromosomal Factors Account for Colistin and Tigecycline Resistance

**Figure 3:**
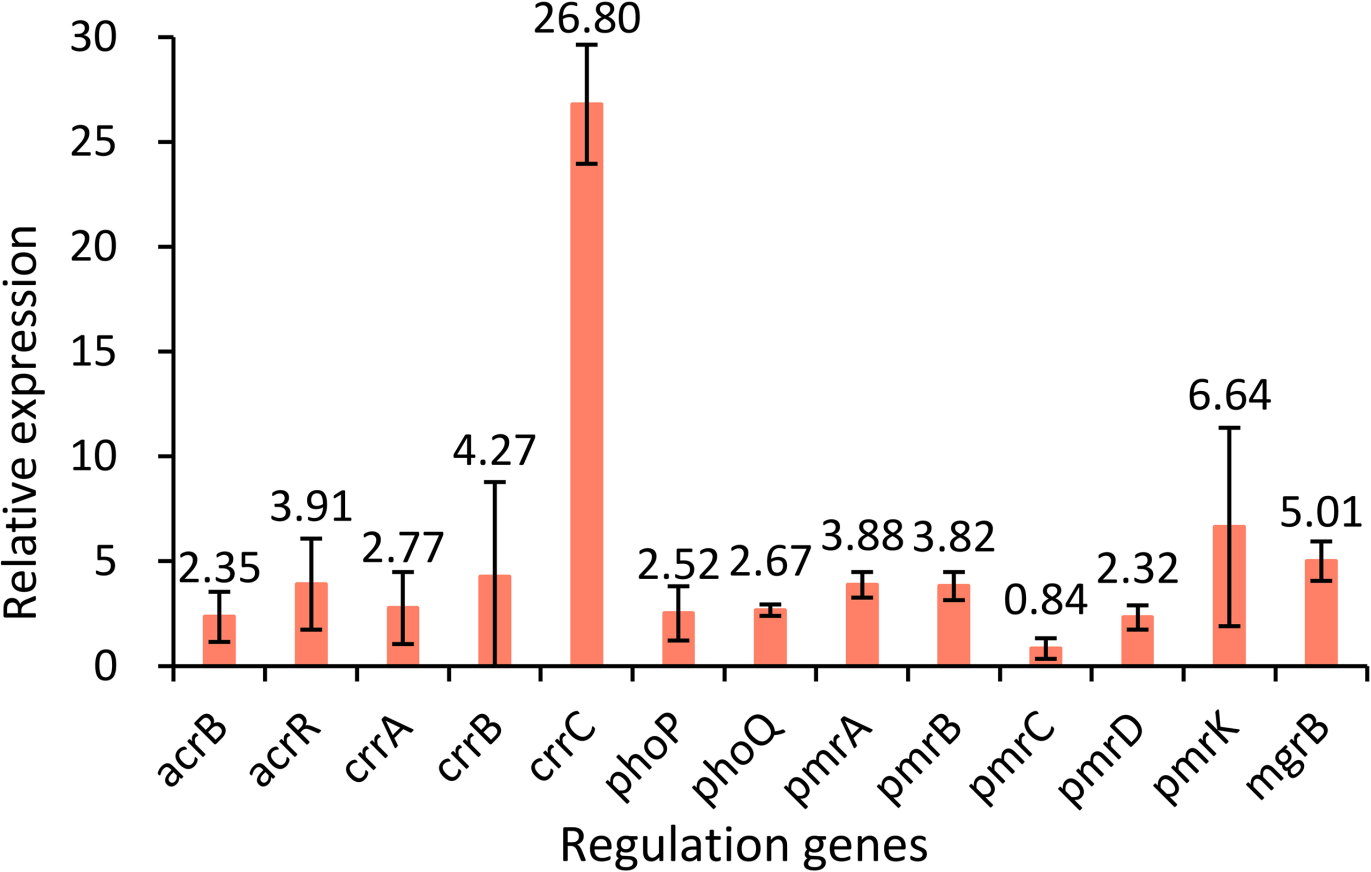
Relative expression levels of the *acrB*, *acrR*, *crrA*, *crrB*, *crrC*, *phoP*, *phoQ*, *pmrA*, *pmrB*, *pmrC*, *pmrD*, *pmrK* and *mgrB* genes in strain KP32558 compared with those in the antibiotic-susceptible *K. pneumoniae* strain ATCC13883. Values are the means and the standard deviations from three independent experiments.

Two-component systems PmrA/PmrB, PhoP/PhoQ and CrrA/CrrB, and the regulator *mgrB* gene were analyzed to identify the potential mutations involved in colistin resistance. Genes in *K. pneumoniae* strain MGH 78578 (CP000647) were used as controls. The results showed that strain KP32558 contained amino acid substitution in MgrB (I45F), PmrA (D86E), PmrB (T246A and G345E) and CrrB (C68S and V193G). PROVEAN was used for predicting the functional effect of the amino acid substitution, indicating that 3 mutations in MgrB, PmrA and CrrB (V193G) were deleterious (have a damaging effect on protein function), while the other 4 mutations were neutral (**Table 2**). These mutations might partly contribute to colistin resistance in *K. pneumoniae* strain KP32558. We examined the expression of the regulatory genes *phoP*, *phoQ*, *pmrA*, *pmrB*, *pmrC*, *pmrD*, *pmrK*, *crrA*, *crrB*, *crrC* and *mgrB*, and the result showed higher expression for all the genes except for *pmrC* (**Figure 3**).

**Table 2:** Mutations in chromosomal genes related to colistin and tigecycline resistance for *K. pneumoniae* KP32558.

Antibiotics	Gene names	Position	Nucleotide mutations	Amino acid change	PROVEAN results
Colistin	*mgrB*	133	A→T	I45F	Deleterious
	*pmrA*	258	T→G	D86E	Deleterious
	*pmrB*	736	A→G	T246A	Neutral
		1034	G→A	G345E	Neutral
	*crrB*	202	T→A	C68S	Neutral
		578	T→G	V193G	Deleterious
Tigecycline	*acrR*	362	G→-	Truncated	Deleterious
	*ramR*	422	T→C	I141T	Neutral

The regulatory genes *ramR*, *marR*, *soxR* and *acrR* of KP32558 were investigated for mutation using *K. pneumoniae* strain MGH 78578 as a negative reference. An amino acid substitution was found in RamR (I141T), which had a neutral effect on the function. AcrR acts as a local transcriptional repressor of the AcrAB efflux pump, the full length of the *acrR* gene in wild-type *K. pneumoniae* isolate is 651 bp. A single-base deletion happened at position 362 of gene *acrR*, resulting in the premature termination of translation at amino acid 123, which could be associated with resistance to tigecycline (Sheng et al., 2014). We examined the expression of a*crR* and *acrB* genes, showing higher expression for both genes, which confirmed the function of AcrR (**Figure 3**).

### Growth Curves of *K. pneumoniae* KP32558 and Transconjugants

Compared to a carbapenem-resistant and hypervirulent *K. pneumoniae* isolate KP22937 (SAMN17245924) and a standard strain *K. pneumoniae* ATCC 700603, KP32558 had a slower growth rate although without statistical difference. While the growth of transconjugant 1 (MCR-8.2) and transconjugant 2 (NDM-5) was almost indistinguishable from that of *E. coli* J53 (**Figure S1**).

## Discussion

The widespread of CRKP has aroused great attention worldwide. Once these strains are resistant to colistin and tigecycline, limited treatment options are available. In this study, we identified an NDM-5-producing *K. pneumoniae* isolate from a lung transplant patient, which simultaneously carried the plasmid-encoded *mcr-8.2* gene and chromosomal gene-mediated resistance to colistin and tigecycline. This represents the rare report of *K. pneumoniae* clinical isolate with multiple resistance mechanisms to the last-resort antimicrobials.

The *bla*_NDM_ gene might be located on different types of plasmids, such as IncFII, IncIB and IncX3, with IncX3 being the most frequently reported. In our study, the *bla*_NDM_ gene was also identified on the IncX3 plasmid. IncX3 plasmids carrying *bla*_NDM_ gene are widely found in *Enterobacterales*, including *E. coli*, *K. pneumoniae*, *Proteus* spp. and *Klebsiella aerogenes* (Zhang et al., 2016; Tian et al., 2020), hinting its wide transmission.

Since the *mcr-8.1* gene from pig-derived *K. pneumoniae* strain KP91 was first reported in 2018, multiple *mcr-8* variants have been identified, ranging from *mcr-8.2* to *mcr-8.5* (Sun et al., 2020). Currently, *K. pneumoniae* producing both MCR-8 and NDM has emerged, such as *mcr-8.1* and *bla*_NDM-1_ (animal origin), *mcr-8.2* and *bla*_NDM-1_ (human origin), *mcr-8.1* and *bla*_NDM-5_ (human origin), and *mcr-8.5* and *bla*_NDM-5_ (animal origin) (Wang et al., 2018; Ma et al., 2020a; Sun et al., 2020; Pei et al., 2021). In our study, we also identified the *K. pneumoniae* isolate co-harboring *mcr-8.2* and *bla*_NDM-5_ in the lung transplantation ward, and should be alerted to its in-hospital spread.

Comparative genomic analysis was conducted to elucidate the genetic characteristics of *mcr-8.2*-carrying plasmid pKP32558-2-mcr8. This plasmid had two replicons, an intact IncFII(K) and a truncated IncQ1. Besides, eight of the ten *mcr-8.2*-carrying plasmids obtained from the GenBank database also had IncFII(K) type replicons, and the other two plasmids carried other IncF type replicons (**Figure 2**). This was consistent with the previous report that the IncF type plasmids are widely distributed in clinically relevant *Enterobacterales* isolates, and contribute to the fitness of bacterial host by providing antimicrobial resistance and virulence determinants (Johnson and Nolan, 2009). IncQ1 type plasmids are efficient antimicrobial resistance determinants among Gram-negative bacteria (Martins et al., 2020). The truncated IncQ1 replicon in the plasmid pKP32558-2-mcr8 was also observed in the other two plasmids, pMCR8_020135 and pMCR8_095845, indicating that these three plasmids may share a common ancestor. Phylogenic analysis of these *mcr-8.2*-carrying plasmids showed that plasmids pKP32558-2-mcr8, pMCR8_020135, pMCR8_095845 and pVNCKp115 were clustered into the same clade. At the same time, the latter three plasmids can match different parts of pKP32558-2-mcr8, indicating that there is a close genetic relationship between them.

MLST analysis showed that the *K. pneumoniae* strain KP32558 was assigned to ST656, a rare ST type and firstly reported in China in 2012 (Wang et al., 2012). The frequently detected carbapenemase in these bacteria was KPC-2 (Liu et al., 2021) and NDM-1 (Li et al., 2020). Our study represents the first report of the emergence of NDM-5 and MCR in ST656 *K. pneumoniae* clinical isolate. The other 10 *mcr-8.2*-carrying plasmids came from bacteria with diverse ST types, including ST1, ST11, ST395, ST1770 and ST3410, suggesting the intra-species spread of mcr-8.2. It was noticeable that pMCR8_020135 and pMCR8_095845 were carried by ST1 *K. pneumoniae* strains, there was only one allele difference between ST656 and ST1, and they belonged to the same clonal complex. These results strengthened our hypothesis that the three plasmids share a common ancestor.

It is believed that without the antibiotic pressure, the acquisition of resistance genes in plasmids will impose fitness costs on their host (Andersson and Hughes, 2010). However, our recent study has shown that the growth of the transconjugant was almost indistinguishable from that of recipient *E. coli* EC600 (Zhao et al., 2021), and another study also showed that up to 75.9% (22/29) *Enterobacterales* strains did not produce fitness costs after obtaining the IncX3 plasmid (Ma et al., 2020b). The above results suggested that the acquisition of IncX3 plasmid might not confer a fitness cost to the host and then facilitate the rapid dissemination of the plasmid. In this study, the *bla*_NDM-5_-carrying IncX3 plasmid pKP32558-5-ndm5 could be transferred to *E. coli* J53 and the transconjugant had a similar growth curve compared with *E. coli* J53, indicating that strain KP32558 probably acquired the resistance to carbapenems by obtaining the *bla*_NDM-5_-carrying and self-transmissible plasmid.

The MIC values for colistin in *mcr-8*-carrying isolates are usually between 4 and 32 mg/L (Wang et al., 2018; Hadjadj et al., 2019). While in our study, the MIC of *K. pneumoniae* strain KP32558 and transconjugant 1 were 256 and 4 mg/L, respectively, hinting that this strain had other colistin resistance mechanisms in addition to the *mcr-8.2* gene. According to a previous report, chromosomal mutations in genes encoding *phoP/phoQ*, *pmrA/pmrB* and c*rrA/crrB* TCSs and mutations in the *mgrB* gene can confer resistance to colistin in *K pneumoniae* (Poirel et al., 2017). In isolate KP32558, a total of six amino acid substitutions were identified in MgrB, PmrA, PmrB and CrrB, among which the substitutions of I45F in MgrB, D86E in PmrA and V193G in CrrB were deleterious according to the PROVEAN tool. The D86E mutation has been described previously (Samuelsen et al., 2017), while the other two mutations are reported for the first time. PmrAB can directly regulate the *pmrHFIJKLM* operon and *pmrC* gene, MgrB negatively regulates genes *phoPQ* by inhibiting the phosphorylation of PhoQ, PhoPQ directly regulates *pmrHFIJKLM* operon and indirectly regulates *pmrC* through PmrD and PmrAB, mutations of *crrB* gene could induce the higher expression of *crrC*, which positively regulates the expression of *pmrHFIJKLM* operon through PmrAB (Cheng et al., 2018). For strain KP32558, we detected higher expression of *crrC*, *pmrA* and *pmrB* genes, but not *pmrC*, which was consistent with the above report. The higher expression of *crrC* gene can be the result of the mutation of *crrB* and the higher expression of *phoP, phoQ and pmrD* was related to the *mgrB* mutation. However, considering the deleterious mutation in *pmrA* and no change in the expression of *pmrC*, we proposed that mutation in *mgrB* may partly contribute to the higher colistin resistance of strain KP32558 through PhoPQ. In addition, missense mutations of *crrB* also lead to increased expression of H239_3064, a putative RND-type efflux pump, leading to higher resistance to colistin (Cheng et al., 2018). We cannot rule out the role of *crrB* mutation in strain KP32558. These results revealed that the *mcr-8.2* gene and chromosomal TCS may have synergistic effects in mediating bacterial resistance to colistin, as reported in a previous study (Wu et al., 2020). Some more experiments, such as the complementation experiment are needed to verify the resistance mechanism of colistin.

High-level expression of efflux pump is the main mechanism of bacterial resistance to tigecycline (Cheng et al., 2020). Mutation in local transcriptional repressor *acrR* and global transcriptional activator *ramA* could result in the overexpression of the AcrAB efflux pump (Rosenblum et al., 2011). One previous study reported 26 tigecycline-nonsusceptible *K. pneumoniae* isolates, among which 23 (88.5%) had mutations in *ramR*, and 2 of the remaining 3 isolates contained a mutation in *acrR* and had a tigecycline MIC of 4 mg/L (Sheng et al., 2014). In this study, an amino acid substitution in RamR (I141T) was found in *K. pneumoniae* strain KP32558, and this mutation had a neutral effect on its function, as consistent with a previous report (Wang et al., 2015). In addition, we also found a single-base deletion at position 362 of *acrR*, which resulted in the premature termination of transcription and showed a deleterious effect on the function. Therefore, we speculated that the mutation in the *acrR* gene may contribute to the resistance of KP32558 to tigecycline.

Infections caused by multidrug-resistant *K. pneumoniae* can cause serious consequences for patients, especially those with compromised immune systems. We previously reported a *K. pneumoniae* isolate KP22937 with an NDM-5-producing plasmid and a hypervirulent plasmid, which caused the death of a lung transplant patient (Zhao et al., 2021). Fortunately, in this study, although pan-drug resistant *K. pneumoniae* isolates were isolated twice in BALF, the bacteria did not cause serious infections in this lung transplant patient and was eliminated without using specific antibiotics. Several mechanisms may contribute to the lower pathogenicity of KP32558. First, no classic hypervirulent genes were found in this strain, which limited its *in-vivo* dissemination. Second, the previous report showed that plasmid carriage often imposes a reduction in the fitness to the host (San Millan and Maclean, 2017). In this study, KP32558 carried 5 plasmids, growth kinetics assay showed that KP32558 had a slower growth rate compared to the standard strain *K. pneumoniae* ATCC 700603 and the *K. pneumoniae* strain KP22937 we previously reported. Although the plasmids carrying NDM-5 or MCR-8.2 did not impose fitness costs on the recipient bacteria, the other 3 plasmids might play a role.

In conclusion, we report an MCR-8.2 and NDM-5-producing pan-drug resistant ST656 *K. pneumoniae* isolate recovered from the BALF specimen of a lung transplant patient. The *mcr-8.2* gene was located on a hybrid plasmid containing IncFII(K) and IncQ1 composition replicons, which may play synergistic effects together with chromosomal mutations in mediating colistin resistance. Efflux pump repressor mutations were also found and may involve in the tigecycline resistance of this *K. pneumoniae* isolate, suggesting that this bacterium has evolved and acquired a variety of complex resistance mechanisms to the last-resort antimicrobials. Therefore, close surveillance is urgently needed to monitor the prevalence of this clone.

## Data Availability Statement

The datasets presented in this study can be found in online repositories. The names of the repository/repositories and accession number(s) can be found in the article/**Supplementary Material**.

## Ethics Statement

Permission for using the information in the medical records of the patient and the *K. pneumoniae* isolates for research purposes was granted by the Ethics Committee of the China-Japan Friendship Hospital (2019-164-K113).

## Author Contributions

JZ, ZL, YZ, XL, and BL collected the clinical and laboratory data. JZ, BL and BC made substantial contributions to conception and design, drafted, reviewed, and edited the manuscript. All authors contributed to the article and approved the submitted version.

## Funding

This work was supported by the National Natural Science Foundation of China [grant number 82102456]; Chinese Academy of Medical Sciences (CAMS) Innovation Fund for Medical Sciences [grant number CIFMS 2020-I2M-2-013 and CIFMS 2021-I2M-1-030].

## Conflict of Interest

The authors declare that the research was conducted in the absence of any commercial or financial relationships that could be construed as a potential conflict of interest.

## Publisher’s Note

All claims expressed in this article are solely those of the authors and do not necessarily represent those of their affiliated organizations, or those of the publisher, the editors and the reviewers. Any product that may be evaluated in this article, or claim that may be made by its manufacturer, is not guaranteed or endorsed by the publisher.

## Supplementary Material

Supplementary Figure 1 S1-PFGE profiles **(A)** and growth curves **(B)** of *K. pneumoniae* clinical strain, recipient bacterium *E. coli* J53 and transconjugants.
