# Supplementary figures and images for: Convergence of MCR-8.2 and Chromosome-Mediated Resistance to Colistin and Tigecycline in an NDM-5-Producing ST656 Klebsiella pneumoniae Isolate From a Lung Transplant Patient in China
Source: Front Cell Infect Microbiol. 2022 Jul 11;12:922031. doi: 10.3389/fcimb.2022.922031 (PMC9310643; doi:10.3389/fcimb.2022.922031)

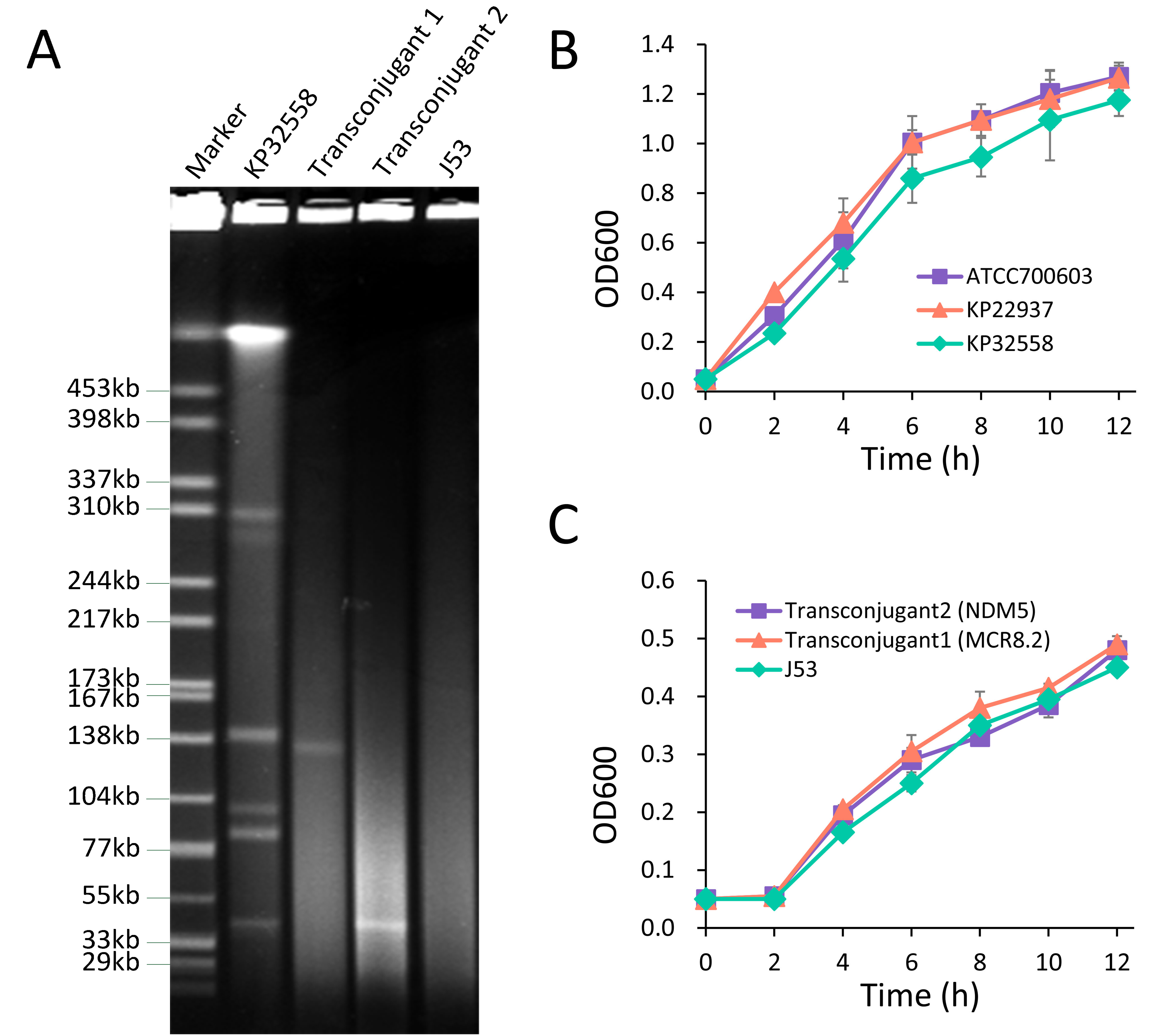

Supplement: Supplementary Figure 1 — S1-PFGE profiles (A) and growth curves (B) of K. pneumoniae clinical strain, recipient bacterium E. coli J53 and transconjugants. [file Image_1.tif]
