# Supplementary material for: Convergence of MCR-8.2 and Chromosome-Mediated Resistance to Colistin and Tigecycline in an NDM-5-Producing ST656 Klebsiella pneumoniae Isolate From a Lung Transplant Patient in China
Source: Front Cell Infect Microbiol. 2022 Jul 11;12:922031. doi: 10.3389/fcimb.2022.922031 (PMC9310643; doi:10.3389/fcimb.2022.922031)
Supplement: Supplementary file 2 [file Table_1.docx]

Table S1. Primers for qRT-PCR

| Primers | Sequence | Length (bp) | Source |
| --- | --- | --- | --- |
| RT-acrB-F | 5’-TCCTCAAATGGCGACTCCAC-3’ | 153 | This study |
| RT-acrB-R | 5’-AACGCTAATCCCTTGCTGCT-3’ |  |  |
| RT-acrR-F | 5’-GTTTTCGCAGCAAGGCGTAT-3’ | 194 | This study |
| RT-acrR-R | 5’-CTGAGAGTGGATCGTTGGGG-3’ |  |  |
| RT-crrA-F | 5’-TCGTCGCAGAAGACGATGAT-3’ | 126 | This study |
| RT-crrA-R | 5’-CAGGCTTGTTCAGACGGGTA-3’ |  |  |
| RT-crrB-F | 5’-CGGGTGGAATGAACGAAGGT-3’ | 93 | This stydy |
| RT-crrB-R | 5’-GTGCAACGACCAGACAGGTA-3’ |  |  |
| RT-crrC-F | 5’-CATTCGGTCTGGTTTTAGTGTTTATACA-3’ | ND | (Cheng et al., 2016) |
| RT-crrC-R | 5’-AAACATAAAGGTAGTCCGCAAAAGA-3’ |  |  |
| RT-phoP-F | 5’-GATCAAGCTGACCGCCTTTG-3’ | 112 | This study |
| RT-phoP-R | 5’-CAGTTCGGCATCCGGGTAAA-3’ |  |  |
| RT-phoQ-F | 5’-GATCACTTTTGTCGGCGAGC-3’ | 92 | This study |
| RT-phoQ-R | 5’-CTTCCACGAACTCCAGGCAA-3’ |  |  |
| RT-pmrA-F | 5’-GTCGCGGCTGATGATGAAAG-3’ | 101 | This study |
| RT-pmrA-R | 5’-CTTCCAGGGTATTGGTCGCC-3’ |  |  |
| RT-pmrB-F | 5’-GGTGGTCTGCCAGCTGATAA-3’ | 99 | This study |
| RT-pmrB-R | 5’-TGGTTGTTGTGCCCTTCGAT-3’ |  |  |
| RT-pmrC-F | 5’-CTCTCGCCTCGTTCCTGAA-3’ | 140 | (Haeili et al., 2017) |
| RT-pmrC-R | 5’-CGGAGTGGTGTCGAGGATA-3’ |  |  |
| RT-pmrD-F | 5’-GCGGGAAGGGGATAAACTCA-3’ | 113 | This study |
| RT-pmrD-R | 5’-TCCAACGCTCGCTGCTATAA-3’ |  |  |
| RT-pmrK-F | 5’-GGTGTATGCGATTGGCACCTA-3’ | 132 | (Haeili et al., 2017) |
| RT-pmrK-R | 5’-AGCAGCACGTAGCCCAGTAT-3’ |  |  |
| RT-mgrB-F | 5’-ACGGTGGGTTTTACTGATAGTC-3’ | 81 | This study |
| RT-mgrB-R | 5’-TCCTGGTCGCACATTACGTT-3’ |  |  |
| RT-rpoB-F | 5’-AAATCACCCAAGGCGACGAT-3’ | 119 | This study |
| RT-rpoB-R | 5’-ACCCTTGTTACCGTGACGAC-3’ |  |  |

Cheng, Y.H., Lin, T.L., Lin, Y.T., and Wang, J.T. (2016) Amino Acid Substitutions of CrrB Responsible for Resistance to Colistin through CrrC in Klebsiella pneumoniae. *Antimicrob Agents Chemother* 60: 3709-3716.doi:10.1128/AAC.00009-16

Haeili, M., Javani, A., Moradi, J., Jafari, Z., Feizabadi, M.M., and Babaei, E. (2017) MgrB Alterations Mediate Colistin Resistance in Klebsiella pneumoniae Isolates from Iran. *Front Microbiol* 8: 2470.doi:10.3389/fmicb.2017.02470
