# Supplementary material for: Convergence of MCR-8.2 and Chromosome-Mediated Resistance to Colistin and Tigecycline in an NDM-5-Producing ST656 Klebsiella pneumoniae Isolate From a Lung Transplant Patient in China
Source: Front Cell Infect Microbiol. 2022 Jul 11;12:922031. doi: 10.3389/fcimb.2022.922031 (PMC9310643; doi:10.3389/fcimb.2022.922031)
Supplement: Supplementary file 3 [file Table_2.docx]

Table S2. Resistance genes in *K. pneumoniae* isolate KP32558 against resfinder database

| Resistance gene | Position | Phenotype |
| --- | --- | --- |
| *fosA* | chromosome | Fosfomycin resistance |
| *oqxB* | chromosome | Disinfectant resistance |
| *bla*_SHV-132_ | chromosome | Beta-lactam resistance |
| *oqxA* | chromosome | Disinfectant resistance |
| *aadA3* | plasmid 1 | Aminoglycoside resistance |
| *aadA1* | plasmid 1 | Aminoglycoside resistance |
| *aph(3'')-Ib* | plasmid 1 | Aminoglycoside resistance Alternate name; aph(3'')-Ib |
| *cmlA1* | plasmid 1 | Phenicol resistance |
| *sul3* | plasmid 1 | Sulphonamide resistance |
| *aac(3)-IV* | plasmid 1 | Aminoglycoside resistance |
| *aph(4)-Ia* | plasmid 1 | Aminoglycoside resistance |
| *aph(6)-Id* | plasmid 1 | Aminoglycoside resistance Alternate name; aph(6)-Id |
| *aph(3')-Ia* | plasmid 1 | Aminoglycoside resistance |
| *mph(E)* | plasmid 1 | Macrolide resistance |
| *msr(E)* | plasmid 1 | Macrolide, Lincosamide and Streptogramin B resistance |
| *armA* | plasmid 1 | Aminoglycoside resistance |
| *sul1* | plasmid 1 | Sulphonamide resistance |
| *bla*_DHA-1_ | plasmid 1 | Beta-lactam resistance AmpC-type |
| *qnrB4* | plasmid 1 | Quinolone resistance |
| *aph(6)-Id* | plasmid 1 | Aminoglycoside resistance Alternate name; aph(6)-Id |
| *tet(A)* | plasmid 2 | Tetracycline resistance |
| *floR* | plasmid 2 | Phenicol resistance |
| *catA2* | plasmid 2 | Phenicol resistance |
| *aadA16* | plasmid 2 | Aminoglycoside resistance |
| *mcr-8* | plasmid 2 | Warning: gene is missing from Notes file |
| *aph(3')-Ia* | plasmid 2 | Aminoglycoside resistance |
| *aac(3)-IId* | plasmid 2 | Aminoglycoside resistance |
| *bla*_TEM-1A_ | plasmid 2 | Beta-lactam resistance Alternate name; blaTEM-1 |
| *sul2* | plasmid 2 | Sulphonamide resistance |
| *aph(3'')-Ib* | plasmid 2 | Aminoglycoside resistance Alternate name; aph(3'')-Ib |
| *aph(6)-Id* | plasmid 2 | Aminoglycoside resistance Alternate name; aph(6)-Id |
| *mph(A)* | plasmid 2 | Macrolide resistance |
| *sul1* | plasmid 2 | Sulphonamide resistance |
| *qnrB6* | plasmid 2 | Quinolone resistance |
| *dfrA27* | plasmid 2 | Trimethoprim resistance |
| *ARR-3* | plasmid 2 | Rifampicin resistance |
| *aac(6')-Ib-cr* | plasmid 2 | Fluoroquinolone and aminoglycoside resistance |
| *oqxA* | plasmid 4 | Disinfectant resistance |
| *oqxB* | plasmid 4 | Disinfectant resistance |
| *bla*_TEM-1B_ | plasmid 4 | Beta-lactam resistance Alternate name; RblaTEM-1 |
| *aph(3')-IIa* | plasmid 4 | Aminoglycoside resistance |
| *fosA3* | plasmid 4 | Fosfomycin resistance |
| *bla*_CTX-M-55_ | plasmid 4 | Beta-lactam resistance |
| *bla*_NDM-5_ | plasmid 5 | Beta-lactam resistance |
